# Subjectively and objectively assessed social and physical environmental correlates of preschoolers’ accelerometer-based physical activity

**DOI:** 10.1186/s12966-017-0577-9

**Published:** 2017-11-06

**Authors:** Michael Eichinger, Sven Schneider, Freia De Bock

**Affiliations:** 1 Mannheim Institute of Public Health, Social and Preventive Medicine, Medical Faculty Mannheim, Heidelberg University, Ludolf-Krehl-Strasse 7-11, 68167 Mannheim, Germany; 2 Department of Pediatrics, University Medicine Mannheim, Heidelberg University, Theodor-Kutzer-Ufer 1-3, 68167 Mannheim, Germany

**Keywords:** Physical activity, Family-level correlates, Community-level correlates, Physical environment, Built environment, Social environment, Preschoolers, Accelerometry, Heart rate monitoring

## Abstract

**Background:**

Overweight and low levels of physical activity (PA) in preschoolers are major public health concerns. However, to date only few studies have investigated subjective *and* objective correlates of PA across different socioecological domains in preschoolers. We therefore simultaneously investigate associations between preschoolers’ objectively measured leisure-time PA and a comprehensive set of subjective and objective potential PA correlates across the behavioral, social and physical environmental domains on both family- and community-level.

**Methods:**

In this cross-sectional study time spent in moderate-to-vigorous PA (MVPA) and total PA (TPA) were measured by combined accelerometry and heart rate monitoring in 735 3–6 year-old children from 52 preschools in Southern Germany. Family- and community-level potential correlates of PA from different domains (behavioral, social and physical environmental) were subjectively (i.e. by parent proxy-report) and objectively assessed. Their associations with PA on weekend days and weekday afternoons were tested by covariate-adjusted multilevel regression models.

**Results:**

While none of the objective social and physical environmental factors showed associations with PA, subjective parental traffic safety perceptions were positively associated with MVPA and TPA on weekends. Also, preschoolers’ participation in organized sports was positively correlated with MVPA (on weekends) and TPA (both on weekends and weekday afternoons).

**Conclusion:**

Subjective traffic safety perceptions and participation in organized sports, an indicator and a result of parental support towards PA – i.e. subjective parental perceptions of environmental factors and family-level correlates which are more proximal to preschoolers - might be more central to PA in preschool age than objectively assessed community-level environmental features which tend to be more distal correlates. If replicable, targeting parental perceptions of environmental factors and parental support for PA in preschool age might be powerful leverages for public health policy.

**Electronic supplementary material:**

The online version of this article (10.1186/s12966-017-0577-9) contains supplementary material, which is available to authorized users.

## Background

Childhood overweight is a major public health concern that affects approximately 20% of children in Europe [1, 2]. Adverse health effects of childhood overweight and obesity include cardiovascular, metabolic and psychosocial health problems [3–8]. In the past, efforts to prevent childhood obesity have mainly focused on individual-level behavior change, with limited effects [9]. Therefore, child health promotion has recently broadened its focus to social and physical environmental potential correlates of obesity [9–11].

Insufficient physical activity (PA), a key determinant of childhood obesity, is widespread among preschoolers [12], with adverse PA behaviors tracking from early childhood to adulthood [13, 14]. Therefore, evidence-informed PA promotion based on detailed knowledge about PA correlates in preschool age is paramount.

While there is a large body of literature on correlates of PA in older children and adolescents [15–19], only a limited number of predominantly cross-sectional studies with often small sample sizes is available for preschool age [20] (with [21] (*n* = 2173) [22] (*n* = 1248) being notable exceptions). To date systematic reviews have only been able to identify a very limited number of consistent associations between PA and its correlates in this age group (e.g. obese parents, preschool location) [20, 23, 24]. In addition, only a few longitudinal studies have investigated determinants of PA in preschoolers so far [25]. These studies mainly used small convenience samples restricting external validity and predominantely assessed a limited number of potential PA correlates (e.g. gender, age, season, parental behaviors) [26–28].

Taken together, our understanding of the complex associations between preschoolers’ PA and its potential correlates is still hampered. Three analytical approaches have been called for that might move the field ahead: Firstly, the socioecological framework emphasizing the multidimensionality of PA correlates has become a well established theoretical model guiding PA research [29]. Recent studies started to concurrently investigate multiple relevant variables across the social as well as physical environmental domains [21, 22, 30]. But more studies that deliberately include potential correlates across the whole spectrum of domains in the socioecological model (i.e. demographic and biological, cognitive and emotional, behavioral, social and cultural, and physical environmental) have been called for in a recent review [24]. Secondly, both the socioecological framework and empirical research emphasize that correlates of preschoolers’ PA operate at different (hierarchical) levels, such as the family-level (e.g. parental PA [28, 31, 32]) and the community-level (e.g. community-level socioeconomic status (SES) [33]). Therefore, including potential correlates pertaining to different levels in a simultaneous analysis seems important. Thirdly, given the widely found divergence between subjective perceptions and objective environmental measurements (e.g. regarding traffic insecurity [34]), supplementing survey-based parental environmental perceptions with objective administrative routine data presents a unique opportunity to increase our understanding of PA correlates in preschool age.

The aim of this study was thus to investigate associations between preschoolers’ accelerometry/heart rate monitoring-based leisure-time PA and a comprehensive set of subjective and objective potential PA correlates across the behavioral, social and physical environmental domains on both family- and community-level in a large sample of German preschoolers.

## Methods

### Setting and participants

In this cross-sectional study (i) family-level (ii) preschool-level (iii) village/city-level as well as (iv) county-level data were used, i.e. the data were clustered at 3 levels. All preschoolers attending the same preschool, that lived in the same village/city or the same county had the same data on all preschool, village/city and county-level potential correlates, respectively (mean numbers of preschoolers with the same data for community-level potential correlates range from 13.9–29.6, lower bound of ranges: 3, upper bound: 128). A *county* in Germany (Kreis) comprises several cities and/or villages. On average 1.4 preschools were nested in one village/city (median: 1, range: 1–4) and 3.5 were nested in a county (median: 2, range: 1–15). Table 1 gives an overview of the levels of measurement of all potential correlates.

**Table 1:**
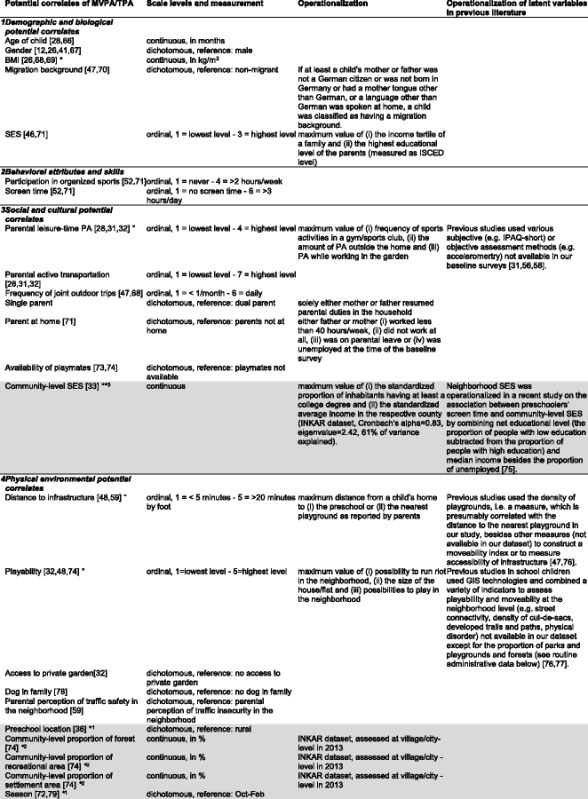
List of potential correlates of moderate-to-vigorous and total physical activity, their measurement properties and operationalization [12, 26, 28, 31-33, 36, 41, 46, 47, 48, 52, 56, 58, 59, 66–79]

Domain classification was adapted from Sallis et al. [44]. No potential correlates belonging to the domain *psychological, cognitive and emotional potential correlates* were assessed in this study. We assessed both family-level (white) and community-level potential correlates (grey) that were measured subjectively (i.e. by parent proxy-report) or objectively (marked with an asterisk *). Among the community-level potential correlates variables were measured at preschool^1^, village/city^2^ or county-level^3^. As the results of 4-level mixed models did not change qualitatively, we collapsed the preschool, village/city and county-levels into one level, called *community-level* in the entire paper. Only 2-level models distinguishing family-level from community-level potential correlates are presented in this paper. Latent variables are marked with a °.

*BMI* body mass index, *ISCED* International Standard Classification of Education, *MVPA* moderate-to-vigorous physical activity, *PA* physical activity, *SES* socioeconomic status, *TPA* total physical activity

Family–level and preschool-level data were based on the baseline measurements of two concurrently implemented cluster-randomized controlled trials in 52 preschools in the German federal state of Baden-Württemberg [35–37]. With a preschool attendance rate of >90% [38] and study preschools being representative for the German preschool system [36], the recruited sample was representative of the population of preschoolers in Germany. Moreover, 80% of all eligible children were recruited further strengthening external validity.

All children aged 3–6 years who were enrolled in one of the 52 preschools were eligible. 1134 were recruited and participated in the baseline measurements of the two intervention studies. Informed written consent was obtained in advance from the parents of all recruited children. The research was approved by the Ethics Committee of the Medical Faculty Mannheim, Heidelberg University (2008-275 N-MA).

The mean age of the preschoolers in our study was slightly higher than in comparable studies due to differences in the German preschool system. After daycare (0–3 years) which is attended by approximately a quarter of children only, kindergarten (3–6 years) is the main educational facility in Germany during the early years. Preschool in the German context is defined as the last year of kindergarten. It is characterized by exercises integrated into everyday kindergarten practices with only a minor focus on math and reading compared to other countries. While our sample was drawn from kindergartens, we used the term *preschool* in order to comply with the predominant nomenclature in the PA literature.

Data collection was conducted between September 2008 and March 2009 and comprised individual and family-level (accelerometry/heart rate data and data on potential correlates assessed by parent proxy-reports) as well as preschool-level data (e.g. preschool location). These data were merged with data on village/city-level and county-level socioeconomic and built environmental factors based on official statistics of the federal government and provided by the *Federal Institute for Research on Building, Urban Affairs and Spatial Developm*ent (INKAR dataset 2013, http://www.bbsr.bund.de).

When merging child-level data with community-level potential correlates, the private addresses of children were approximated by preschool addresses due to the unavailability of addresses for a majority of preschoolers. In a subsample of 370 preschoolers for whom individual addresses were available, the ZIP codes of the preschoolers’ addresses and those of the preschools agreed in 95% of cases. As sensitivity analyses showed qualitatively comparable results using 4-level models (i.e. taking into account the clustering of the data at 3 levels; results not shown), we used 2-level models for our analyses, collapsing the preschool, village/city and county-levels into one level, henceforth called *community-level*.

### Outcomes: Objective moderate-to-vigorous and total physical activity

Outcomes of the study were time spent in moderate-to-vigorous PA (MVPA) and total PA (TPA) outside of preschool. As previous studies in preschoolers documented different levels of PA on weekend days versus weekdays [23, 39–41] with potentially different factors influencing preschoolers’ PA, we analyzed separate models for weekends and weekday afternoons. Preschool characteristics were shown to be associated with PA during preschool time [42] suggesting different PA correlates for leisure-time PA versus PA at preschool. As we specifically wanted to investigate correlates of leisure-time PA, i.e. PA outside of the preschool context, we used PA during weekday afternoons and during weekends as outcomes. Accordingly, only children that exclusively attended preschool during mornings (9 am – 1 pm) were included in the weekday afternoon sample, comprising the time between 1 and 9 pm. The weekend sample included Saturdays and Sundays from 7 am – 9 pm for all children, as in Germany children only attend preschools during the week. Actiheart devices (Actiheart software version 13.1.4., CamNtech, Cambridge, UK) were used for accelerometry and heart rate monitoring for up to six consecutive days including two weekend days (epoch length 15 s, continuous 24 h recording). Time spent in MVPA was assessed by combined accelerometry and heart rate monitoring using previously validated cut-offs (boys: accelerometry >118 counts/15 s and heart rate > 134 beats/min; girls: accelerometry >105 counts/15 s and heart rate > 138 beats/min) [43]. TPA was assessed by mean accelerometry counts per 15 s from 1 to 9 pm and 7 am – 9 pm, respectively. Recordings had to last at least 4 h/day to be considered valid and children had to have recordings for Saturday *and* Sunday and at least three weekday afternoons to be included in the final analyses, respectively.

### Potential correlates of physical activity behaviors

**Fig. 1:**
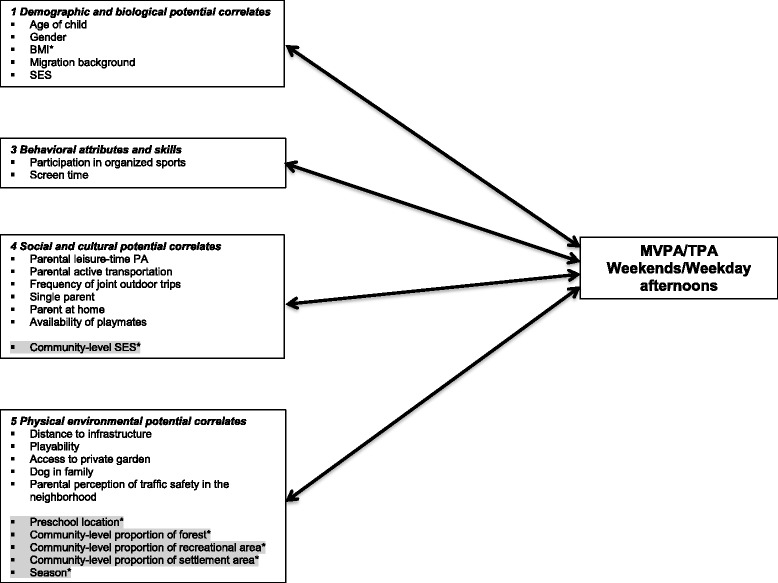
Potential correlates of moderate-to-vigorous and total physical activity. The subjectively (i.e. by parent proxy-report) or objectively (marked with an asterisk) assessed variables in the four domains were concurrently tested in multilevel regression models. Both family- (white) and community-level potential correlates (grey) were included in the analyses. Domain classification was adapted from Sallis et al. [44]. No potential correlates belonging to the domain *psychological, cognitive and emotional potential correlates* were assessed in this study. BMI, body mass index; ISCED, International Standard Classification of Education; MVPA, moderate-to-vigorous physical activity; PA, physical activity; SES, socioeconomic status;. TPA, total physical activity

Potential correlates of PA (Fig. 1) were identified from comprehensive literature reviews [20, 23, 44, 45] and then grouped into four domains of the socioecological framework presented by Sallis et al. [44]: (1) demographic and biological, (2) behavioral, (3) social and cultural as well as (4) physical environmental potential correlates. Psychological, cognitive and emotional potential correlates (i.e. the fifth domain in the framework of Sallis et al.) were not available. Potential correlates were assessed at the family- and community-level and deliberately included both subjectively (parent proxy-report, e.g. parental perceptions) as well as objectively measured (i.e. data from routine administrative datasets) potential correlates (Table 1). Potential correlates were included in the multilevel models if previous studies had shown them to be associated with preschoolers’ PA (references are presented in Table 1). Details (measurement, operationalization and scale level) on all potential correlates included in the final models are provided in Table 1. To reduce the number of potential correlates in the models, underlying latent variables were identified by correspondence (ordinal variables) and factor analyses using principal component analysis and applying an orthogonal rotation (continuous variables). The operationalization of the latent variables was based on previous research (Table 1). Cronbach’s alpha was calculated to assess the internal consistency of the extracted continuous latent variable.

### Statistical analysis

After descriptive and bivariate analyses, associations between potential correlates and objectively assessed MVPA and TPA on weekdays and weekends were tested by a single covariate-adjusted multilevel regression model each. The four models included all potential correlates and a random intercept to account for the clustered data structure (ICC = 0.05 and ICC = 0.07 for MVPA and TPA, respectively).

Point estimates for the regression models were expressed in minutes/weekend day and minutes/weekday afternoon for MVPA and mean counts/15 s for TPA, respectively. Only cases with complete data were considered in the analyses. Introducing quadratic terms into the models for the variables age and BMI (to reach an approximately Gaussian distribution of the residuals) did not change the results qualitatively (except for BMI being additionally associated with MVPA and TPA on weekday afternoons compared to the compact specifications). Therefore, only models without quadratic terms are presented in the results section. All statistical analyses were conducted in 2016 using STATA (version 13, StataCorp, College Station, United States).

## Results

### Participants

**Fig. 2:**
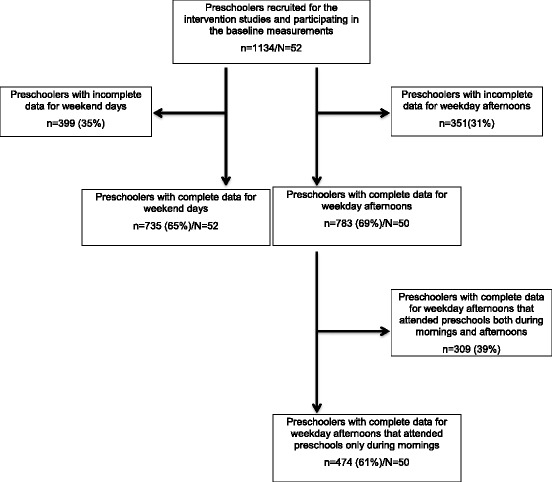
Flow diagram of sample sizes of the multilevel models. n, number of children included in the respective sample/analysis; N, number of preschools included in the respective sample/analysis

In our study, 735 and 783 preschoolers (see flow diagram in Fig. 2) from 52 and 50 preschools had complete data for weekends and weekday afternoons, respectively, and hence could be included in the multilevel models. Preschoolers with complete data attending preschool both during mornings and afternoons (39%) were excluded from further analyses in the weekday models, leaving 474 preschoolers in the weekday afternoon models (Fig. 2). Child and community-level sample characteristics are presented in Table 2. Mean daily recording times were 12.68 ± 3.01 and 7.42 ± 1.29 h for weekend days and weekdays, respectively. On average, children spent 3.9% of the weekend waking time and 4.3% of weekday afternoons in MVPA. We observed differences in the mean time spent in MVPA during weekends between preschoolers included in the final models and those excluded due to incomplete data (subsample in final models: 32.55 ± 20.55 min/weekend day, subsample excluded due to incomplete data: 26.00 ± 21.53 min/weekend day, respectively; *p* < 0.001). Moreover, differences in the distribution of several potential correlates were observed between preschoolers included in the final models and those with incomplete data (Additional file 1: Table S1).

**Table 2:**
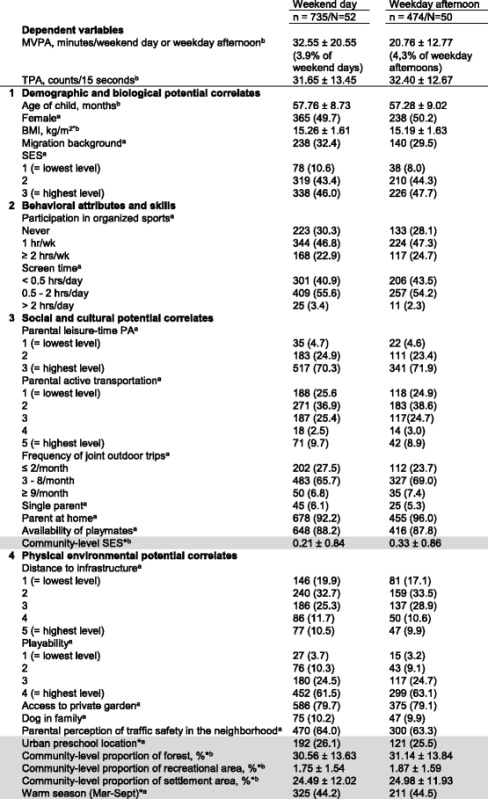
Distribution of child, family and community characteristics in the weekend and weekday afternoon samples

Domain classification was adapted from Sallis et al. [44]. For the domain *psychological, cognitive and emotional potential correlates* in the framework of Sallis et al. no factors were assessed in this study. We assessed both family-level (white) and community-level potential correlates (grey) that were measured subjectively (i.e. by parent proxy-report) or objectively (marked with an asterisk *). Values are ^a^n (%) and ^b^mean ± SD. For ease of display, categories of ordinal variables were merged (if available, based on national recommendations [80]) and hence do not match with the categories as used in the multilevel models (Table 3). Due to rounding errors percentages do not always add up to 100%. MVPA was measured in minutes/weekend day and minutes/weekday afternoon, respectively. TPA was measured in mean accelerometer counts/15 s during weekend days and weekday afternoons, respectively. A weekend day and a weekday afternoon comprised the period between 7 am - 9 pm and 1–9 pm, respectively.

*BMI* body mass index, *MVPA* moderate-to-vigorous physical activity, *n* number of children in the respective sample, *N* number of preschools in the respective sample, *PA* physical activity, *SES* socioeconomic status, *TPA* total physical activity.

### Multilevel models for moderate-to-vigorous and total physical activity

The covariate-adjusted multilevel linear regression models for both weekends and weekday afternoons showed significant associations between the outcome variables (MVPA and TPA) and family-level behavioral correlates as well as subjective parental physical environmental perceptions (Table 3). While certain differences in relations are evident, the overall pattern of associations was comparable across MVPA and TPA (Table 3). In contrast, none of the objective social and physical environmental variables from the community-level routine administrative dataset (community-level socioeconomic status (SES), proportions of forest, recreational area as well as settlement area) or the preschool location (rural versus urban) were associated with any of the outcomes (Table 3).

**Table 3:**
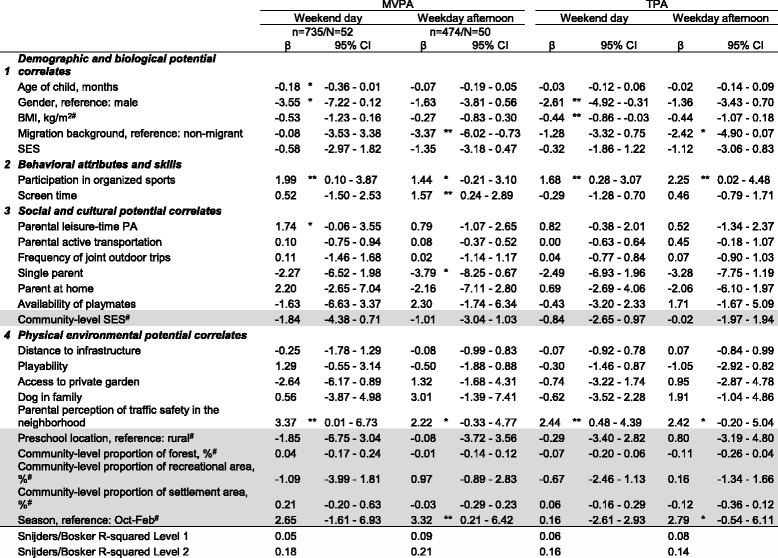
Associations of potential correlates with moderate-to-vigorous and total physical activity

The subjectively (i.e. by parent proxy-report) or objectively (marked with a hashtag ^#^) assessed variables in the four domains were concurrently tested in the multilevel regression models. Both family- (white) and community-level potential correlates (grey) were included in the analyses. Domain classification was adapted from Sallis et al. [44]. No potential correlates belonging to the domain *psychological, cognitive and emotional potential correlates* were assessed in this study. MVPA and TPA were measured in minutes/weekend day or minutes/weekday afternoon and mean accelerometer counts/15 s, respectively. A weekend day and a weekday afternoon comprised the period between 7 am - 9 pm and 1–9 pm, respectively. If units of independent variables are not otherwise specified they are measured on an ordinal scale (details on measurement, scale levels and operationalization are provided in Table 1).

*BMI* body mass index, *CI* confidence interval, *MVPA* moderate-to-vigorous physical activity, *n* number of children included in the respective analysis, *N* number of preschools included in the respective analysis, *PA* physical activity; SES, socioeconomic status, *TPA* total physical activity.

*P < 0,1, **P < 0,05.

In the behavioral domain, a child’s participation in organized sports was positively associated with MVPA on weekends (β = 1.99 min/weekend day [95% CI 0.10–3.87]). For TPA a positive association was documented for both the weekend (β = 1.68 mean counts/15 s [0.28–3.07]) and the weekday afternoon sample (β = 2.25 mean counts/15 s [0.02–4.48]). In addition, MVPA during weekday afternoons was positively associated with subjective (i.e. parent proxy-report) screen time (β = 1.57 min/weekday afternoon [0.24–2.89]), but no such association was found for any of the other outcomes (Table 3).

Within the group of family-level social correlates, subjective parental leisure-time PA showed a trend towards significance for MVPA in the weekend sample (β = 1.74 min/weekend day [−0.06–3.55]), but no such finding was documented for the other models.

Within the family-level physical environmental domain, subjective parental perceptions of traffic safety in the neighborhoods were consistently associated with the PA outcomes. For weekends, parental perceptions were positively associated with both MVPA (β = 3.37 min/weekend day [0.01–6.73]) and TPA (β = 2.44 mean counts/15 s [0.48–4.39]). Within the group of objective community-level physical environmental correlates, season was significantly associated with MVPA in the weekday sample only (β = 3.32 min/weekday afternoon [0.21–6.42]).

In addition, demographic and biological covariates such as gender and migration background showed significant associations with preschoolers’ MVPA and TPA (see Table 3 for details).

## Discussion

This study assessed associations between preschoolers’ objectively measured leisure-time PA and a comprehensive set of subjective (i.e. by parent proxy-report) and objective potential PA correlates across the behavioral, social and physical environmental domains on both family- and community-level (Fig. 1) in a large sample of German preschoolers.

None of the objective community-level social and physical environmental potential correlates were associated with objectively assessed leisure-time PA. In contrast, for the family-level behavioral domain (child participation in organized sports) as well as the subjective (i.e. by parent proxy-report) family-level physical environmental domain (parental perception of neighborhood traffic safety) significant associations with MVPA and TPA were observed. These main findings were consistently seen in both the weekend and weekday afternoon samples.

No associations between PA and the objective community-level social and physical environmental factors from routine administrative datasets (community-level SES and proportions of forest, recreational as well as settlement area) – i.e. distal potential correlates - were observed. To date only few studies have investigated associations between objective community-level potential correlates and PA in preschool age [21, 22]. Our results are in line with a study in Dutch preschoolers showing fewer associations between subjectively assessed outdoor play and distal physical environmental correlates (i.e. correlates that are less central to a person, e.g. greenness of the neighborhood) than proximal correlates (e.g. presence of a private garden) [21].

The lack of associations observed in our study might partly be due to the measurement of objective environmental correlates at the community-level, i.e. all preschoolers attending preschools in one community had the same data on the objective community-level potential correlates. As the area in which a substantial proportion of preschoolers’ PA happens tends to be quite small [46], our assessment might not have been detailed enough to capture relevant yet subtle differences in the PA environment. An objective assessment of the environment at the level of the individual child (e.g. by creating and analyzing child-level buffers in GIS), possibly compared to community-level measures, might move the field ahead.

In our study none of the subjective (i.e. parent proxy-reported) family-level physical environmental correlates apart from parental perceptions of the neighborhood traffic safety were associated with PA. In contrast, a small number of North-American and Australian studies identified several physical environmental correlates of PA in preschoolers (predominantly assessed by parent proxy-report, e.g. number of playgrounds within walking distance [47], distance to the next park [48], presence of public open spaces and the number of recreational facilities in public open spaces [46]). In contrast to these results distance to infrastructure (i.e. to formal play areas like the next playground) were not associated with PA in our study. This might be explained by differences between North-American and European land-use planning traditions (e.g. increased availability of informal play areas like sidewalks in European settings) [49–51]. Moreover, the variables playability and access to private garden, mainly capturing informal opportunities to play, were not associated with PA in our study either. This is in contrast to a recent study in Dutch preschoolers that showed a positive association between the presence of sidewalks, i.e. one type of informal opportunity to play, and outdoor play [22]. The result of the Dutch study highlights the potential beneficial effect of informal play areas already in preschool age which should be further investigated in future studies.

Subjectively assessed regular participation in organized sports was positively associated with all PA outcomes in our study. The literature however remains inconclusive: A study on children aged 5–12 years (*n* = 518) documented a positive association between participation in organized sports and parent proxy-report leisure-time PA [52], while a recent review including two smaller studies (*n* = 214; *n* = 347) reported no association between formal sports participation and PA [20]. Possible explanations for these divergent results might be different methods of PA assessment (objective measurement by accelerometry/heart rate monitoring versus direct observation), different settings (overall leisure-time PA versus PA during time spent in childcare versus PA at home) or differing degrees of control for potential confounders [47, 53] .

There is a growing body of literature documenting a consistent positive association between parental PA and PA behaviors in preschool age [54–57]. In the present study, however, we only found a non-significant association between subjectively assessed (i.e. parent reported) parental leisure-time PA, a family-level social environmental correlate, and MVPA on weekends with no significant associations for any of the other outcomes. One reason could be our joint assessment of leisure-time PA for mothers and fathers: One study in preschoolers [28] as well as a review including studies in older children [45] showed that only PA levels of fathers were a correlate of children’s PA whereas the mothers’ PA was mostly unrelated. For studies that did not separate PA of parents no associations were observed [45].

Alternatively, participation in organized sports as a potential proxy-measure for parental support might mediate the relationship between parental PA and their children’s PA levels as shown by Loprinzi et al. [58]. As effect estimates were not affected by excluding participation in organized sports from our models (data not shown), substantial mediating effects seem unlikely but can not be precluded. Future studies might want to further assess potential indirect and direct effects in the relationship between parental and offspring PA.

The positive association between preschoolers’ leisure-time PA and subjective parental perceptions of neighborhood traffic safety, a family-level physical environmental correlate, is in line with Timperio et al. [59], documenting that children whose parents perceived neither lights nor crossings within the neighborhood were less likely to actively commute to school. In contrast, other empirical evidence did not show an association between subjective perceptions of neighborhood safety and PA behaviors [60]. These mixed results might be due to the assessment of different dimensions of safety (traffic versus crime) and different types of PA (overall leisure-time PA versus active transportation versus outdoor play).

Recent evidence shows that negative impacts of parental safety concerns on offspring PA are not confined to preschool age but are also observed in older children’s independent mobility (10 to 12 year-olds) [61]. This might be due to a persistent negative association between parental safety concerns and offspring unstructured PA and play across different periods of childhood. Interventions already during the preschool period might therefore yield the largest benefits and should be prioritized in future research.

While some studies identified road safety as a valid parental concern substantiated by child pedestrian accident statistics [62], others showed no correlation between objective road insecurity and subjective parental concerns [34]. Future studies should aim at elucidating which dimensions of neighborhood safety (traffic and/or crime) are most important for which type of PA and whether objectively measured and subjectively perceived traffic safety concur.

Surprisingly, we found that subjectively assessed (i.e. by parent proxy-report) screen time, one of the family-level behavioral correlates, was positively associated with MVPA on weekday afternoons, but not with any other outcome. However, also previous literature on screen time and its association with PA remains inconclusive [20, 23], with lack of sedentary behavior during screen-based activities in young children being one potential explanation [63].

The lack of association between most PA outcomes and age, BMI, migration status and SES is in line with a majority of studies included in recent reviews [20, 23, 24]. The results for these demographic and biological correlates did not change once all community-level potential correlates had been excluded from the models in sensitivity analyses (results not shown).

Estimated mean magnitudes of associations between MVPA and its correlates, particularly parental perceptions of neighborhood traffic safety and participation in organized sports, ranged from approximately 1.5–3.5 minutes per weekend day and weekday afternoon, respectively. While small in absolute terms, the coefficients represent a relative increase of time spent in MVPA per weekend day and weekday afternoon of 7–10%, respectively. Given increasing evidence that particularly MVPA (vs. lower intensity PA) is associated with health gains in children and youth [64], the magnitude of associations observed in our study might well be relevant in public health terms.

### Strengths and limitations

Strengths of our study are the objective PA measurement by combined accelerometry and heart rate monitoring and the deliberate use of linear mixed models to account for the clustered data structure. In contrast, many studies on potential correlates of PA relied on proxy-report measures of PA and disregarded the clustered data structure in statistical analyses [45].

Our study adds to the limited number of recent studies simultaneously investigating multiple potential correlates of preschoolers’ PA from different domains of the socioecological model (e.g. [21, 22] (outcome: subjectively assessed outdoor play) [30] (outome: PA counts per minute)). In contrast, older studies often reported only bivariate results or confined themselves to investigating a few correlates (mean 3.9 correlates/study, range: 1–14) [20]. By concurrently assessing a multidimensional set of potential correlates, the complexity of factors influencing preschoolers’ PA is approached in a much more comprehensive way and the most effective intervention leverages might be better isolated.

Moreover, the present paper adds to the limited body of literature on social and physical environmental potential correlates of preschoolers’ PA in European countries [20–22]. The majority of studies to date have reported findings from North-American and Australian samples [20, 23] – countries with clearly distinct land-use planning traditions and built environments [49–51].

Despite these strengths, the study has several limitations. Firstly, like most studies investigating potential correlates of PA [20, 23, 24], the present study uses a cross-sectional design precluding any causal inferences. Future studies should select longitudinal study designs to advance our understanding of the correlates of PA. Secondly, we observed significant differences with regards to mean time spent in MVPA during weekends and the distribution of several potential PA correlates between preschoolers included in the final models and those excluded due to incomplete data. A larger proportion of preschoolers excluded from the analyses due to incomplete data had migration background and was from a low SES background and spent less time in MVPA during weekends. While 80% of eligible children were recruited, this presumably non-random pattern of missing data might have biased our results and might therefore limit the generalizability of our findings. The observed pattern of missing data and non-response is common in many studies that rely on parental consent and proxy-report. Using administrative routine data at the level of the preschoolers (e.g. objectively assessed BMI from school entrance examinations) and objective measures of PA correlates might minimize this source of selection bias in future studies. Thirdly, despite the objective assessment of a limited number of potential PA correlates, a substantial subset of potential PA correlates was still measured via parent proxy-report. Future research might benefit from supplementing or even substituting these subjective measures with objective approaches to minimize measurement error (e.g. assessment of distances to playgrounds and preschools by GPS/GIS-based approaches) [45, 46]. Fourthly, we were only able to assess parental perceptions with regards to neighborhood traffic safety. As parental perceptions might shape preschoolers’ PA behaviors beyond this narrow area, future studies should assess parental perceptions of a broader set of neighborhood characteristics (e.g. neighborhood greenness, insecurity due to crime). Fifthly, we were only able to include a limited set of family-level physical environmental potential correlates (e.g. access to private garden, presence of a dog in families). Future studies investigating the association between both indoor (e.g. indoor PA equipment) and outdoor (e.g. size of garden) home physical environmental correlates and objectively measured PA should use novel technologies to objectively assess the home environment (e.g. wearable cameras) where possible [65]. Lastly, the present dataset only included limited information on parental characteristics (e.g. parental modeling, support). With accumulating evidence that parental characteristics might be key correlates of PA in preschool age [28, 31, 54–56, 58, 59], gathering detailed information on parents in future studies seems necessary.

## Conclusions

This paper investigated associations between preschoolers’ objectively measured leisure-time PA and a comprehensive set of subjective (e.g. parental perceptions) and objective potential PA correlates across the behavioral, social and physical environmental domains on both family- and community-level in a large representative sample of preschoolers in a German context. While none of the objective social and physical environmental factors showed significant associations with PA, subjective parental perceptions of neighborhood traffic safety as well as participation in organized sports, possibly mirroring parental support towards PA, were positively associated with MVPA and TPA. Family-level correlates and subjective parental perceptions of environmental factors – i.e. correlates that are more proximal to preschoolers – might be more central to PA in preschool age than objectively assessed community-level environmental features, i.e. more distal correlates. Future longitudinal and intervention studies should therefore include these correlates to further strengthen the evidence base. If our findings can be replicated, targeting parental perceptions of environmental factors and parental support for PA in preschool age might be powerful leverages for future public health policy at the municipal and district level.

## Additional files

Additional file 1: **Table S1.** Sample characteristics of the subsample included in the final models and the subsample excluded from the final models due to incomplete data. (XLSX 35 kb)

## Abbreviations

BMI: Body mass index
ISCED: International Standard Classification of Education
MVPA: Moderate-to-vigorous physical activity
PA: Physical activity
SES: Socioeconomic status
TPA: Total physical activity
